# Overground Gait Training With a Wearable Robot in Children With Cerebral Palsy: A Randomized Clinical Trial

**DOI:** 10.1001/jamanetworkopen.2024.22625

**Published:** 2024-07-22

**Authors:** Ja Young Choi, Seung Ki Kim, Juntaek Hong, Hankyul Park, Shin-seung Yang, Dongho Park, Min-Keun Song

**Affiliations:** 1 Department of Rehabilitation Medicine, Chungnam National University Hospital, Daejeon, Korea; 2 Department and Rehabilitation Medicine, Yongin Severance Hospital, Yonsei University College of Medicine, Yongin, Korea; 3 Department and Research Institute of Rehabilitation Medicine, Yonsei University College of Medicine, Seoul, Korea; 4 Department and Rehabilitation Medicine, Seoul Rehabilitation Hospital, Seoul, Korea; 5 George W. Woodruff School of Mechanical Engineering, Georgia Institute of Technology, Atlanta; 6 Institute for Robotics and Intelligent Machines, Georgia Institute of Technology, Atlanta; 7 Department of Physical and Rehabilitation Medicine, Chonnam National University Medical School and Hospital, Gwangju, Korea

## Abstract

**Importance:**

Cerebral palsy (CP) is the most common developmental motor disorder in children. Robot-assisted gait training (RAGT) using a wearable robot can provide intensive overground walking experience.

**Objective:**

To investigate the effectiveness of overground RAGT in children with CP using an untethered, torque-assisted, wearable exoskeletal robot.

**Design, Setting, and Participants:**

This multicenter, single-blind randomized clinical trial was conducted from September 1, 2021, to March 31, 2023, at 5 rehabilitation institutions in Korea. Ninety children with CP in Gross Motor Function Classification System levels II to IV were randomized.

**Intervention:**

The RAGT group underwent 18 sessions of RAGT during 6 weeks, whereas the control group received standard physical therapy for the same number of sessions during the same period.

**Main Outcome and Measures:**

The primary outcome measure was the Gross Motor Function Measure 88 (GMFM-88) score. Secondary outcome measures were the GMFM-66, Pediatric Balance Scale, selective control assessment of the lower extremity, Pediatric Evaluation of Disability Inventory–Computer Adaptive Test (PEDI-CAT), 6-minute walking test scores (distance and oxygen consumption), muscle and fat mass via bioelectrical impedance analysis, and gait parameters measured via 3-dimensional analysis. All assessments were performed for all patients at baseline, at the end of the 6-week intervention, and after the 4-week follow-up.

**Results:**

Of the 90 children (mean [SD] age, 9.51 [2.48] years; 49 [54.4%] male and 41 [45.6%] female) in the study, 78 (86.7%) completed the intervention, with 37 participants (mean [SD] age, 9.57 [2.38] years; 19 [51.4%] male) and 41 participants (mean [SD] age, 9.32 [2.37] years; 26 [63.4%] male) randomly assigned to the RAGT and control groups, respectively. Changes in the RAGT group significantly exceeded changes in the control group in GMFM-88 total (mean difference, 2.64; 95% CI, 0.50-4.78), GMFM-E (mean difference, 2.70; 95% CI, 0.08-5.33), GMFM-66 (mean difference, 1.31; 95% CI, 0.01-2.60), and PEDI-CAT responsibility domain scores (mean difference, 2.52; 95% CI, 0.42-4.63), indicating independence in daily living at postintervention assessment. At the 4-week follow-up, the RAGT group showed significantly greater improvements in balance control (mean difference, 1.48; 95% CI, 0.03-2.94) and Gait Deviation Index (mean difference, 6.48; 95% CI, 2.77-10.19) compared with the control group.

**Conclusions and Relevance:**

In this randomized clinical trial, overground RAGT using a wearable robot significantly improved gross motor function and gait pattern. This new torque-assisted wearable exoskeletal robot, based on assist-as-needed control, may complement standard rehabilitation by providing adequate assistance and therapeutic support to children with CP.

**Trial Registration:**

CRIS Identifier: KCT0006273

## Key Points

**Question:**

Is robot-assisted gait training with wearable exoskeletal robots more effective than conventional physical therapy in children with cerebral palsy (CP)?

**Findings:**

This randomized clinical trial of 90 children with CP found significant improvements in gross motor function, balance control, and gait pattern among children receiving robot-assisted gait training.

**Meaning:**

These findings suggest that an untethered, torque-assisted, wearable exoskeletal robot, based on assist-as-needed control, is effective in children with CP.

## Teaser

This randomized clinical trial of children with cerebral palsy compares overground robot-assisted gait training using an untethered, torque-assisted, wearable exoskeletal robot with standard physical therapy.

## Introduction

Cerebral palsy (CP) is the most common neuromotor disorder in children, limiting walking and daily activity.^1^ Damage to the developing brain in CP leads to abnormal motor experience due to altered neurologic function, including weakness, spasticity, loss of motor control, and limited coordination.^2^ The consequential abnormal sensorimotor experiences and movement restrictions adversely affect musculoskeletal changes and cortical disorganization, resulting in a vicious circle.^2^

Children with CP have various gait impairments that significantly affect their daily activities and social integration.^3^ Therefore, a key therapeutic goal in children with CP is to enhance walking ability.^4^ Treadmill training, partial body weight support gait training, and goal-directed training are known to enhance motor function in children with CP.^5^ With recent technological advancements, robot-assisted gait training (RAGT) offers constant patterns of repetitive, high-intensity, and goal-oriented training—the basic principle of effective rehabilitation. However, a recent meta-analysis revealed weak and inconsistent evidence of RAGT effects in children with CP.^6^ Most devices included in the meta-analysis were based on treadmill-tethered, trajectory-controlled robots. Further research should explore various types of robotic systems to establish more robust clinical evidence for RAGT.

Regarding assistive control strategy, 2 types of RAGT devices can be distinguished: torque control or gait trajectory controllers. The trajectory-controlled robot operates on a kinematic basis along a predefined gait path and set joint angle, regardless of the residual power, which is typically used for complete motor impairment, whereas the torque-controlled model adjusts the amount of force applied at each joint to aid movement based on the user’s effort and motion, requiring active participation from the patient.^7^ Most patients with CP can generate some effort at the major joints of the lower limbs, despite varying degrees of motor impairment. As for traditional training methods with gait robots, there are treadmill-tethered robots and overground robots with a wearable suit. The most developed pediatric RAGT devices are treadmill-tethered trajectory-controlled or end-effector robots. However, to our knowledge, no multicenter large-scale randomized clinical trials exist on the effects of overground wearable exoskeleton RAGT in children with CP.

The recently developed torque-assisted wearable RAGT device allows training on various terrains, including overground terrain, ramps, and stairs. This robotic suit can assist joint motion based on assist-as-needed control, potentially promoting active participation. Active engagement, generalization of tasks in the real world, and kinematic variability during gait are essential for effective motor learning.^8,9,10^ In contrast, conventional RAGT devices with constant guidance and full support often lead to participants being passive, reducing patient effort and weakening the motor learning effect.^11,12^ Numerous exoskeletons have been developed; however, only a few have been tailored for the pediatric population. Therefore, this study aimed to investigate the effects of overground RAGT using an untethered, torque-assisted, wearable exoskeletal robot in children with CP.

## Methods

### Study Design

This multicenter, prospective, single-blinded randomized clinical trial was conducted from September 1, 2021, to March 31, 2023, at 5 pediatric rehabilitation centers in Korea. The internal review board of each participating hospital approved the study, and the trial was registered with the Clinical Research Information Service. The trial was reported in alignment with the Consolidated Standards of Reporting Trials (CONSORT) reporting guidelines.^13^ Written informed consent was obtained from the patient’s parents. The flowchart of the study is shown in the Figure. The trial protocol can be found in Supplement 1.

**Figure.**
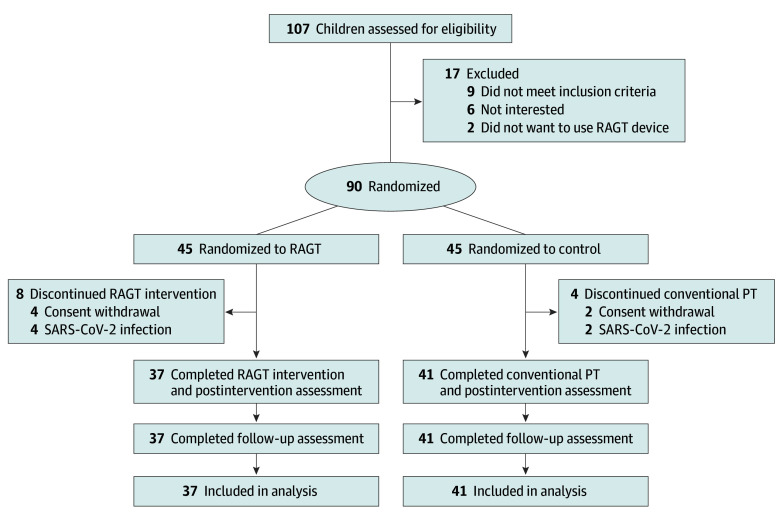
CONSORT Study Flow Diagram PT indicates physical therapy; RAGT, robot-assisted gait training.

### Participants

The study included children with CP aged 6 to 15 years who were 98 to 150 cm tall. Children with gait difficulty who walked with or without assistance at Gross Motor Function Classification System levels II to IV were included in the study. Children were excluded if they had severe intellectual disabilities that precluded understanding simple commands, severe lower limb contractures that interfered with wearing a robotic device, unhealed skin lesions on the lower limbs, a history of orthopedic surgery within the previous 12 months, or chemodenervation within the previous 6 months. Chemodenervation, surgery, or alteration of antispastic medication regimens were prohibited during the study. In total, 90 children were enrolled.

### Randomization and Masking

After the baseline assessments, participants were randomly assigned to the intervention or control group using a centralized web-based randomization system. An independent statistician generated a randomization list at the start of the trial using a computerized R program, version 3.5.1 (R Foundation). To avoid assessment bias, physical therapists blinded to the participants’ group assignments conducted all evaluations.

### Interventions

The experimental group underwent 30 minutes of RAGT, whereas the control group underwent 30 minutes of conventional physical therapy (PT) 3 days per week for 6 weeks. Both groups received the same total number of therapy sessions (18 sessions, 9 hours overall) during the intervention period. The number of training steps per session to assess training intensity was measured using a pedometer in the control group and a robotic device in the RAGT group.

The RAGT device was the Angel Legs M20, size S (Angel Robotics Inc), a powered lower-limb wearable robot designed to assist children with disabilities in improving their walking and lower limb mobility. Equipped with actuators at each hip and knee joint, the device can provide assistive torque according to the gait phase, automatically detected using the combined information from the ground contact sensor, encoders (incremental and absolute) in the actuators, and an inertial measurement unit sensor in a backpack (eFigure 1 in Supplement 2).^14^ Robot-assisted gait training was performed overground in an indoor hospital setting, using an overhead lift or walker for balance, particularly for children with Gross Motor Function Classification System levels of III or IV, as needed. Each training session included the actual walking time in the RAGT (30 minutes), time to put on and take off the robot, rest, and evaluation of adverse events. The control group received conventional PT focused on gait training for 30 minutes per session in a 1-on-1 setting (eAppendix in Supplement 2). Participants who missed more than 10% of the treatment sessions within the 6-week intervention period were dropped from the study.

After the end of the intervention, follow-up assessments were conducted 4 weeks later to investigate the maintenance of the intervention effects. During this period, both groups received standard care, which consisted of a mean of 2 PT sessions weekly, including gait training, tone reduction, balance, and strengthening exercises. Therapies were individually adjusted based on the child’s function (eFigure 2 in Supplement 2).

### Outcome Measures

Functional and kinematic assessments were performed for all patients at baseline (within 72 hours before the intervention), at the end of the 6-week intervention (within 1 week after intervention, posttest 1), and after the 4-week follow-up (22-34 days after intervention, posttest 2) to investigate effect maintenance.

#### Motor Function Assessments

The primary outcome measure was the overall gross motor function measured using the Gross Motor Function Measure (GMFM)-88. The GMFM-88 was selected as the primary outcome measure due to its robust validation and comprehensive ability to assess overall gross motor function changes, crucial for evaluating the impact of gait-focused interventions in CP. The percentage scores of dimensions D (standing ability) and E (walking, running, and climbing abilities), total GMFM-88 score, and GMFM-66 score were used for the analysis. The minimal clinically important difference (MCID) for the GMFM-88 total and GMFM-66 ranged from a 1.0% to 2.0% increase, suggesting a clinically significant improvement in gross motor function.^15,16^

Balance control was assessed using the Pediatric Balance Scale, consisting of 14 items with 0 to 4 points per item and a maximum score of 56 points (with low scores indicating poor balance control and high scores indicating good balance control).^17^ Selective voluntary motor control capacity was quantified using the Selective Control Assessment of the Lower Extremity (SCALE).^18^ A SCALE score was obtained by summing the 0 to 2 points assigned to each of the 5 joints for a maximum of 10 points per limb. For both assessments, higher scores indicated better functioning.

#### Physical Fitness and Participation in Daily Activities

To assess walking endurance and physical fitness, we measured walking distance (in meters) and oxygen consumption for 6 minutes (using the 6-minute walking test [6-minute WT]) with open-circuit spirometry (Kb4,^2^ COSMED USA Inc). Subsequently, the oxygen rate and oxygen costs were calculated. The MCID for the 6-minute WT was 20 to 46 m.^15^ Bioelectric impedance analysis was used to estimate muscle mass and body fat percentage as proportions of total body weight. The Pediatric Evaluation of Disability Inventory–Computer Adaptive Test (PEDI-CAT) was used to assess performance in daily activities. This test measures functional skills in 4 domains, including daily activity, mobility, social cognitive, and responsibility, with 276 items based on the parent or caregiver report.^19^ In our study, the scaled scores (range, 0-100, with 0 indicating low functional ability and 100 indicating high functional ability) of each domain were used for the analysis.

#### Gait Parameters

The participants walked barefoot along a 10-m walkway at a self-selected speed. Participants were fitted with 16 passive reflective markers according to the Helen Hayes marker set. Kinematic and temporospatial data were measured using a computerized 3-dimensional motion analysis system (VICON MX-T10 Motion Analysis System, Oxford Metrics Inc). Spatiotemporal gait parameters, including cadence, walking speed, stride and step width, and single and double supports (percentage of the gait cycle) were used for analysis. The Gait Deviation Index (GDI) was calculated using kinematic data. The GDI quantifies overall gait pathology by providing a single score ranging from 0 to 100, with values closer to 100 indicating a normal gait pattern.

### Statistical Analysis

Sample size calculations were performed using a 2-tailed test with a randomized controlled design. A sample size of 82 was sufficient to detect an effect size of 0.6, power of 90%, and significance level of 5%. The sample size was increased to 90, allowing an anticipated dropout rate of approximately 10%.

A linear mixed-effects regression model was used to compare the efficacy of RAGT with that of conventional PT. We analyzed each outcome separately, with treatment, time, and interaction effects as independent variables. The Mann-Whitney *U* test or independent 2-tailed *t* test was used to compare the extent of improvement between the baseline and postintervention statuses. Mean differences at postintervention and after the 4-week follow-up were estimated by the group × time interaction term, with associated 95% CIs. *P* < .025 indicated statistical significance by Bonferroni-adjusted post hoc analysis. Data were analyzed using SPSS, version 28 (IBM Inc).

## Results

Among 90 participants (mean [SD] age, 9.51 [2.48] years; 49 [54.4%] male and 41 [45.6%] female), 78 (86.7%) completed the intervention. Specifically, 37 participants (mean [SD] age, 9.57 [2.38] years; 18 [48.6%] female and 19 male [51.4%]) were randomly assigned to the RAGT group, and 41 participants (mean [SD] age, 9.32 [2.37] years; 15 [36.6%] female and 26 [63.4%] male) to the control group. Eight children in the RAGT group and 4 in the control group dropped out of the trial owing to consent withdrawal (n = 6) or SARS-CoV-2 infection of participants or parents (n = 6). Demographic characteristics did not differ significantly (Table 1).

**Table 1.** Characteristics of the Participants^a^

Characteristic	RAGT group (n = 37)	Control group (n = 41)
Age, mean (SD), y	9.57 (2.38)	9.32 (2.37)
Sex		
Female	18 (48.6)	15 (36.6)
Male	19 (51.4)	26 (63.4)
GMFCS		
II	14 (37.8)	15 (36.6)
III	15 (40.5)	16 (39.0)
IV	8 (21.6)	10 (24.4)
Involved side		
Unilateral	5 (13.5)	5 (12.2)
Bilateral	32 (86.5)	36 (87.8)
Height, mean (SD), cm	128.96 (16.45)	129.21 (13.17)
Body weight, mean (SD), kg	34.54 (21.70)	30.83 (10.33)

Abbreviations: GMFCS, Gross Motor Function Classification System; RAGT, robot-assisted gait training.

^a^ Data are presented as number (percentage) of participants unless otherwise indicated.

### Robot-Assisted Gait Training

No safety issues were reported, and the experimental group experienced no adverse effects, such as skin lesions, pain, or fatigue, during RAGT. A specially trained physical therapist attached the RAGT device to all the participants. The number of training steps per session significantly differed between the groups (mean [SD] steps per session: RAGT group, 997.6 [124.5] steps; control group, 212.9 [116.6] steps; *P* < .001).

### Motor Function Assessments

The RAGT group showed significant improvements in gross motor function, as measured by the GMFM-88 total (mean difference, 2.64; 95% CI, 0.50-4.78), dimension E (mean difference, 2.70; 95% CI, 0.08-5.33), and GMFM-66 (mean difference, 2.52; 95% CI, 0.42-4.63) scores compared with the control group at the postintervention assessment. Furthermore, the immediate increases in the GMFM-88 total, dimension E, and GMFM-66 scores in the RAGT group in our study were 2.7%, 2.3%, and 1.2%, respectively, compared with baseline, for which the MCID thresholds were achieved after the intervention, indicating clinically significant changes. This improvement was maintained until the 4-week follow-up (Table 2).

**Table 2.** Motor Functional Outcome Measures at Baseline, After Intervention, and at 4-Week Follow-Up

Measure	Least squares mean (SE)		Time group interaction, estimated mean difference (95% CI)
	RAGT group (n = 37)	Control group (n = 41)	
GMFM-88 total			
Baseline	69.99 (3.84)	66.34 (3.93)	NA
Postintervention assessment	72.73 (3.54)^a^	66.68 (3.96)	2.64 (0.50 to 4.78)^b^
4-wk Follow-up	74.03 (3.44)^a^^,^^c^	67.84 (3.85)	2.68 (0.54 to 4.83)^b^
GMFM-88 dimension D			
Baseline	50.24 (5.69)	44.25 (5.58)	NA
Postintervention assessment	52.67 (5.73)^a^	44.84 (5.67)	2.36 (−0.23 to 4.95)
4-wk Follow-up	54.68 (5.58)^a^	45.56 (5.73)	3.48 (0.89 to 6.07)^b^
GMFM-88 dimension E			
Baseline	43.17 (5.34)	36.86 (5.55)	NA
Postintervention assessment	45.50 (5.54)^a^	37.29 (5.61)	2.70 (0.08 to 5.33)^b^
4-wk Follow-up	47.07 (5.48)^a^	38.14 (5.74)	2.93 (0.31 to 5.55)^b^
GMFM-66			
Baseline	59.81 (2.11)	61.97 (2.90)	NA
Postintervention assessment	61.00 (2.14)^a^	63.82 (3.03)^a^	1.31 (0.01 to 2.60)^b^
4-wk Follow-up	61.77 (2.11)^a^	64.41 (2.96)^a^	0.96 (−0.34 to 2.26)
PBS			
Baseline	26.49 (3.38)	24.41 (3.30)	NA
Postintervention assessment	27.89 (3.44)^a^	25.44 (3.37)^a^	0.38 (−1.07 to 1.83)
4-wk Follow-up	28.43 (3.54)^a^	24.87 (3.36)	1.48 (0.03 to 2.94)^b^
SCALE			
Baseline	9.65 (1.07)	9.08 (0.83)	NA
Postintervention assessment	10.53 (1.10)^a^	9.72 (0.89)^a^	0.29 (−0.62 to 1.19)
4-wk Follow-up	10.77 (1.06)^a^	10.00 (0.87)^a^	0.23 (−0.68 to 1.13)

Abbreviations: GMFM, Gross Motor Function Measure; NA, not applicable; PBS, Pediatric Balance Scale; RAGT, robot-assisted gait training; SCALE, Selective Control Assessment of the Lower Extremity.

^a^ *P* < .025 by Bonferroni-adjusted post hoc analysis compared with baseline assessment within the group.

^b^ Positive value indicates that the RAGT group had a greater mean change at that time point compared with the control group.

^c^ *P* < .025 by Bonferroni-adjusted post hoc analysis compared with postintervention assessment within the group.

Improvement of balance control measured by the Pediatric Balance Scale was significantly greater in RAGT group compared with the control group at the 4-week follow-up (mean difference, 1.48; 95% CI, 0.03-2.94). Selective motor control measured using SCALE was improved in both groups, with no significant group differences.

### Physical Fitness and Participation in Daily Activities

The improvement in gait endurance on the 6-minute WT was observed only in the RAGT group at 4-week follow-up, with no significant differences between the groups (Table 3). There were no significant changes in physical fitness measures, including oxygen consumption and muscle and fat mass via bioelectric impedance analysis after intervention in both groups. According to the PEDI-CAT, changes in the responsibility domain were greater in the RAGT group compared with the control group (mean difference, 2.52; 95% CI, 0.42-4.63). The changes in the daily activity (mean difference, 0.58; 95% CI, −0.30 to 1.46), mobility (mean difference, 0.10; 95% CI, −1.98 to 2.18), and social cognitive (mean difference, 0.01; 95% CI, −1.02 to 1.04) domains did not significantly differ between the groups (Table 3).

**Table 3.** Physical Fitness and Participation in Daily Activities at Baseline, After Intervention, and at 4-Week Follow-Up

Measure	Least squares mean (SE)		Time group interaction, estimated mean difference (95% CI)
	RAGT group	Control group	
**Gait endurance**				
6-min WT, m			
Baseline	196.34 (18.89)	166.87 (19.83)	NA
Postintervention assessment	204.69 (18.50)	183.35 (21.65)	−8.13 (−26.06 to 9.79)
4-wk Follow-up	215.04 (19.19)^a^	184.16 (21.59)	1.41 (−16.52 to 19.33)
**Oxygen consumption**				
Oxygen rate, mL/kg/min			
Baseline	18.23 (1.29)	19.11 (1.29)	NA
Postintervention assessment	18.35 (0.92)	19.88 (1.06)	−0.63 (−3.10 to 1.83)
4-wk Follow-up	17.69 (1.15)	21.16 (1.06)	−2.42 (−4.86 to 0.03)
Oxygen cost, mL/kg/m			
Baseline	1.15 (0.31)	0.89 (0.20)	NA
Postintervention	1.28 (0.39)	1.30 (0.38)	−0.18 (−0.86 to 0.50)
4-wk Follow-up	0.85 (0.15)	1.17 (0.30)	−0.49 (−1.17 to 0.18)
**BIA, %**				
Skeletal muscle			
Baseline	37.13 (1.03)	37.35 (0.83)	NA
Postintervention assessment	36.14 (1.03)	36.59 (0.88)	−0.22 (−1.98 to 1.53)
4-wk Follow-up	36.18 (1.02)	36.97 (0.91)	−0.57 (−2.33 to 1.18)
Fat			
Baseline	25.11 (2.03)	24.58 (1.80)	NA
Postintervention assessment	27.27 (2.03)	26.60 (1.78)	0.14 (−3.10 to 3.37)
4-wk Follow-up	27.10 (2.04)	25.90 (1.84)	0.67 (−2.56 to 3.91)
**PEDI-CAT**				
Daily activity			
Baseline	52.62 (0.91)	52.63 (1.05)	NA
Postintervention assessment	53.59 (0.84)^a^	53.02 (1.06)	0.58 (−0.30 to 1.46)
4-wk Follow-up	53.85 (0.92)^a^	53.56 (1.07)^a^	0.28 (−0.61 to 1.16)
Mobility			
Baseline	57.16 (1.18)	55.22 (1.18)	NA
Postintervention assessment	57.68 (1.13)	55.63 (1.20)	0.10 (−1.98 to 2.18)
4-wk Follow-up	57.02 (1.14)	57.29 (1.07)^a^	0.30 (−0.74 to 1.34)
Social cognitive			
Baseline	65.46 (1.07)	64.68 (1.02)	NA
Postintervention assessment	66.05 (1.13)	65.27 (1.01)	0.01 (−1.02 to 1.04)
4-wk Follow-up	66.17 (1.05)	65.07 (1.08)	0.30 (−0.74 to 1.34)
Responsibility			
Baseline	47.51 (1.65)	48.22 (1.19)	NA
Postintervention assessment	48.87 (1.50)	47.05 (1.17)	2.52 (0.42 to 4.63)^b^
4-wk Follow-up	49.66 (1.30)	47.51 (1.18)	3.02 (0.91 to 5.14)^b^

Abbreviations: 6-min WT, 6-minute walking test; BIA, bioelectrical impedance analysis; NA, not applicable; PEDI-CAT, Pediatric Evaluation of Disability Inventory–Computer Adaptive Test; RAGT, robot-assisted gait training; WT, walk test.

^a^ *P* < .025 by Bonferroni-adjusted post hoc analysis compared with baseline assessment within the group.

^b^ Positive values indicate that the RAGT group had a greater mean change at that time point compared with the control group.

### Gait Parameters

Regarding the temporospatial parameters, decrement of step width was significantly greater in the RAGT group compared with the control group, indicating improvement (mean difference, −0.05; 95% CI, −0.08 to −0.01). However, gait speed and stride length did not significantly change (Table 4). Notably, at the 4-week follow-up, the duration of single limb support of the more involved limb increased, whereas double limb support decreased only in the RAGT group, indicating improvement but without significant group differences. Improvement on the GDI was significantly greater in the RAGT group compared with the control group at 4-week follow-up (mean difference, 6.48; 95% CI, 2.77-10.19).

**Table 4.** Gait Analysis at Baseline, After Intervention, and at 4-Week Follow-Up

Measure	Least squares mean (SE)		Time group interaction, estimated mean difference (95% CI)
	RAGT group	Control group	
Cadence			
Baseline	96.79 (7.62)	86.07 (9.84)	NA
Postintervention assessment	92.41 (7.26)	87.48 (9.61)	−5.80 (−14.04 to 2.44)
4-wk Follow-up	93.63 (7.29)	91.03 (8.55)	−7.72 (−16.05 to 0.60)
Walking speed, m/s			
Baseline	0.63 (0.07)	0.54 (0.09)	NA
Postintervention assessment	0.58 (0.06)	0.57 (0.08)	−0.07 (−0.16 to 0.02)
4-wk Follow-up	0.62 (0.07)	0.58 (0.07)	−0.04 (−0.13 to 0.05)
Stride length, m			
Baseline	0.71 (0.05)	0.64 (0.06)	NA
Postintervention assessment	0.71 (0.04)	0.68 (0.05)	−0.04 (−0.12 to 0.03)
4-wk Follow-up	0.75 (0.04)	0.69 (0.05)	−0.01 (−0.09 to 0.07)
Step width, m			
Baseline	0.16 (0.02)	0.12 (0.02)	NA
Postintervention assessment	0.14 (0.01)	0.14 (0.01)	−0.05 (−0.08 to −0.01)^a^
4-wk Follow-up	0.14 (0.01)	0.13 (0.01)	−0.03 (−0.06 to 0.01)
Single support, % (more involved limb)			
Baseline	29.59 (1.66)	30.14 (2.29)	NA
Postintervention assessment	30.31 (1.72)	30.54 (2.69)	0.31 (−2.36 to 2.97)
4-wk Follow-up	32.07 (1.58)^b^	30.05 (2.36)	2.63 (−0.06 to 5.33)
Single support, % (less involved limb)			
Baseline	32.12 (1.85)	29.07 (2.59)	NA
Postintervention assessment	33.07 (1.82)	31.14 (2.50)	−1.12 (−3.63 to 1.40)
4-wk Follow-up	34.10 (1.66)	32.42 (2.66)	−0.35 (−2.89 to 2.19)
Double support, %			
Baseline	33.04 (4.06)	38.62 (5.37)	NA
Postintervention assessment	32.09 (3.70)	34.11 (5.39)	3.56 (−0.72 to 7.84)
4-wk Follow-up	27.83 (3.22)^b^^,^^c^	34.62 (5.30)	−1.35 (−5.67 to 2.98)
Gait Deviation Index			
Baseline	65.55 (2.32)	73.35 (2.24)	NA
Postintervention assessment	66.03 (2.51)	71.26 (2.45)	2.58 (−1.33 to 6.49)
4-wk Follow-up	69.14 (2.35)	70.36 (2.50)	6.48 (2.77 to 10.19)^a^

Abbreviations: NA, not applicable; RAGT, robot-assisted gait training.

^a^ Positive values indicate that the RAGT group had a greater mean change at that time point compared with the control group, whereas negative values indicate that the RAGT group had a significant decrease compared with the control group.

^b^ *P* < .025 by Bonferroni-adjusted post hoc analysis compared with baseline assessment within the group.

^c^ *P* < .025 by Bonferroni-adjusted post hoc analysis compared with the postintervention assessment within the group.

## Discussion

We reported the findings of a large, multicenter trial involving the use of a newly developed torque-assisted wearable robot in children with CP. After a 6-week RAGT training, gross motor function improved in the study group compared with the control group, and the effect was maintained throughout the follow-up. The immediate increases in the GMFM-88 total, dimension E, and GMFM-66 scores in the RAGT group in our study were 2.7%, 2.3%, and 1.2%, respectively, compared with baseline, for which the MCID thresholds were achieved after the intervention, indicating clinically significant changes. Gait pattern, balance control, and participation in daily living also improved after RAGT compared with the control group. No adverse events, such as pain, skin lesions, increased fatigue, or falls, were reported.

Robot-assisted gait training enhances motor function through repetitive and intensive training with a constant gait pattern. A certain threshold for repetition must be crossed to induce effective brain reorganization. This experience-dependent neuroplasticity requires at least 1000 repetitions of the same task to achieve permanent change at the synaptic level.^20^ Herein, the RAGT group reached nearly 1000 steps per training session, 4.7 times the training intensity of the control group.

Although partial weight-bearing treadmill training has been proven effective, the evidence supporting the effectiveness of RAGT remains inconclusive. A recent meta-analysis revealed that RAGT did not confer significantly improved gross motor function, 6-minute WT performance, or gait speed compared with a dose-matched standard of care.^6^ In another meta-analysis that compared RAGT with various control groups,^21^ RAGT was associated with improved gait speed and gross motor function compared with conventional PT but was not superior to treadmill gait training or, when used in combination with conventional PT and RAGT, was not superior to PT alone. The heterogeneity of the RAGT effect may be explained by the following factors: device type, purpose of the device and interacting interface, study design, therapeutic dose with randomized clinical trial design, function of the participants, and outcome measurements.

Active engagement in training is critical for effective neurorehabilitation. During overground gait training, children can navigate and explore within a more natural environment, achieve active neuromuscular engagement, and attain greater stride-to-stride variability.^22^ Additionally, overground RAGT exoskeletons seem more effective for dynamic balance than tethered robots, facilitating appropriate body alignment during weight-shifting movements.^14,23^ The normal gait control mechanism involves maintaining stability in the anterior and lateral directions during forward progression.^24^ Notably, the RAGT used in this study was able to capture step initiation using a ground contact and inertial measurement unit sensor to assist torque according to the gait phase. This intention-capturing feedback mechanism may elicit longer-term effects than passive training, which uses a bottom-up approach. In this study, balance improved in both groups; however, the carryover effect was maintained only in the RAGT group.

Passive rhythmic movement activates the gait center or central pattern generators in the spinal cord.^25^ Compared with the traditional treadmill training or RAGT, this new overground RAGT offering variable gait experience might elicit a higher level of spine and brain neuroplasticity.^26^ Recently, to overcome passive aspects and restricted movement variability of treadmill-tethered RAGT, some devices have offered varying levels of guidance or assistance that may be adjusted based on the patient’s requirements. Compared with treadmill training or end-effector-type RAGT, the RAGT exoskeleton enables more physiologic and reproducible sensorimotor experience in joint angular motion. These effects can be maintained while walking despite removing the device. In this study, gait pattern improved by increasing GDI and single-limb support and decreasing double-limb support. Robot-assisted gait training enables a sufficient stance phase on the side of the affected limb, promoting gait symmetry and reducing the compensatory mechanism during gait by assisting joint movements.

Predefined gait trajectory control is the most common assistive control strategy for exoskeletons. Trajectory-controlled RAGT devices are relatively simple but force the wearer to walk on a reference trajectory, possibly not aligning with their natural walking pattern.^27^ Patients with complete paralysis or severe motor impairment can be trained to walk while wearing a robotic device, whereas movement can be too restricted in patients with incomplete paralysis. Robot-assisted gait training that provides continuous full guidance often makes patients passive and reduces their effort, weakening the motor learning effect in patients with incomplete paralysis,^11,12^ whereas model-based control assists patients with mild to moderate paralysis but requires a precise dynamic model of the human exoskeleton system.^7,27^ The unique feature of the RAGT device in this study is that the torque can be adjusted according to the patient’s residual muscle strength, allowing more active participation, variability of movements, and dynamic gait pattern adaptation.^12^ This robot can offer assistance at various joints at varying levels, which enables customized intervention.

### Limitations

This study had several limitations. First, lower limb movements were assisted only in the sagittal plane. Second, only patients who could use a small RAGT device were included in this study. Additional studies are required to delineate the optimal training parameters and the role of the robot device type according to the patient profile.

## Conclusions

In this randomized clinical trial, overground RAGT using a wearable robot significantly improved gross motor function, balance control, and gait pattern compared with a matched conventional PT group. This new torque-assisted, wearable RAGT benefits children with CP, supporting power-as-needed control, motivating children to explore walking, and providing intensive gait training. These benefits could also apply to children with prewalking motor abilities.

## Notes

Supplement 1.
Trial Protocol

Supplement 2. **eFigure 1.** The Torque-Assisting Wearable Exoskeletal Robot **eFigure 2.** Study Flowchart **eAppendix.** Conventional Physical Therapy (PT) for Control Group

Supplement 3.
Data Sharing Statement
