# Supplementary material for: Overground Gait Training With a Wearable Robot in Children With Cerebral Palsy: A Randomized Clinical Trial
Source: JAMA Netw Open. 2024 Jul 22;7(7):e2422625. doi: 10.1001/jamanetworkopen.2024.22625 (PMC11265136; doi:10.1001/jamanetworkopen.2024.22625)
Supplement: Supplement 1. — Trial Protocol [file jamanetwopen-e2422625-s001.pdf]

Overground gait training using wearable exoskeletal robot in children with cerebral palsy

# : A randomized controlled trial

Principal Investigator

Ja Young Choi, MD, PhD

Chungnam National University

1. Study Title: The effect of overground gait training using wearable exoskeletal robot in children with cerebral palsy: A randomized controlled trial

2. Study Objectives

a. **Study Aims/Objectives/Hypotheses:**

This study was designed to establish the clinical evidence for effect of robot-assisted gait training (RAGT) with wearable exoskeletal robots in children with cerebral palsy. First, we would compare the effect of RAGT with werable robot to conventional physical therapy.

- Purpose : To investigate the effects of overground RAGT in children with CP using an untethered torque-assisted wearable exoskeletal robot.

3. Study sites: Republic of Korea

- Study Site 1: Chungnam National Universtiy Hospital, Deajeon, Republic Korea
- Study Site 2: Yonsei University Health System, Severance Hospital, Seoul, Republic Korea
- Study Site 3: Seoul Rehabilitation Hospital, Seoul, Republic Korea
- Study Site 4: Yongin Severance Hospital, Yonsei University College of Medicine, Yongin, Korea
- Study Site 5: Chonnam National University Medical School & Hospital, Gwangju, Korea

4. Study design: Multicenter, single blind, randomized controlled trial.

This study is designed as a group randomized trial, with measures collected pre and post intervention.

5. Participant selection, Recruitment and Consent Procedures:

a. **Inclusion Criteria:**

1) Children with cerebral palsy
2) GMFCS (Gross Motor Function Classification System) level II, III, IV
3) Patients without cognitive impairment who are able to comply with protocol-required procedure
4) male or female, 6 to 15 years 11months
5) Voluntarily agreed and singed the consent

b. **Exclusion Criteria:**

1) Significant intellectual disabilities, hard to apply study device
2) Skin/ Musculoskeletal deformity, hard to wear study device
3) Etc, The subject who is deemed unsuitable for this study
4) Botulinum toxin therapy in the study limb within 6 months prior to the baseline assessment day or History of treatment with alcohol block or orthopedic surgery in the study limb within 12 months prior to the baseline assessment day

c. **Recruitment Procedures:** Children with cerebral palsy or static brain injury, who visit the 5 rehabilitation institutions in Korea (The Chungnam National University Hospital, Korea coordinated this trial in collaboration with Yonsei University Hospital, Yongin Severance Hospital, Seoul Rehabilitation Hospital, and Chonnam National University Hospital). After screening assessment, and all parents and patients were informed about the purpose and protocol before enrollment. At enrollment, the inclusion and exclusion criteria were applied by performing a detailed review of each patient's medical history.

d. **Consent Procedures:** Child consent will be obtained from parents/guardians of children asked to participate in the study. Program staff will distribute consent letters to parents/guardians of children attending the protocol.

6. Study Procedures:

a. Randomization

After baseline assessment, participants were randomized into either the intervention or the control group through a centralized web-based randomization system. The system assigned patients randomly to the experimental or the control group in a 1:1 ratio. Randomization sequence was generated at the start of the trial using a computerized R program (version 3.5.1., R foundation, Vienna, Austria).

b. Research procedures/tests

Total 90 children with cerebral palsy with gait difficulty who walked with or without assistance were included (45 children will receive only conventional physical therapy, and other 45 children will receive the occupational therapy using video-game assisted device).

The RAGT device was the Angel Legs M20, size S (Angel Robotics Inc., Seoul, Republic of Korea), a powered lower-limb wearable robot with four hip and knee actuators. The robot can provide assistive torque according to the gait phase, automatically detected using the combined information from the ground contact sensor, encoders (incremental and absolute) in the actuators, and an inertial measurement unit sensor in a backpack. RAGT was performed overground in an indoor hospital setting using an overhead lift or walker in children with GMFCS levels III or IV, as needed. Each training session included the actual walking time in the RAGT (30 min), time to put on and take off the robot, rest, and evaluation of adverse events. The control group received conventional PT focused on gait training for 30 min per session in a one-on-one setting.

<Intervention protocol >

- Treatment time/ frequency: 30 minutes of session, Total 18 sessions (3 days/week for 6 weeks)

- RAGT group : 30 minutes of RAGT x 18 sessions

- control group: 30 minutes of conventional PT x 18 sessions

c. Assessment

- Assessment tool:

- Motor function assessments: Gross Motor Function Measure (GMFM)-88, GMFM-66, Pediatric Balance Scale (PBS), Selective Control Assessment of the Lower Extremity (SCALE)
- Physical fitness and participation in daily activities: 6 min walking test (6 minWT), oxygen consumption test, Bioelectric impedance analysis (BIA), Pediatric Evaluation of Disability Inventory-Computer Adaptive Test (PEDI-CAT),
- 3D computerized gait analysis: Kinematic data, Spatiotemporal gait parameters: cadence, walking speed (m/s), stride and step length and width, and single and double supports

- Evaluation plan:

1) pre-intervention, 2) immediately after intervention, 3) 4 weeks after end of intervention

(1) Gross Motor Function Measure (GMFM)-88, 66

- standardized assessment tool that uses 88 tasks scored on five subscales. The scores assigned to each subscale were converted into percentages using the maximum possible score. The scores of dimensions D (standing ability) and E (walking/running/climbing abilities) and a total of 0–100% were used for the analysis. The GMFM-66 is a newer version, which was reduced to 66 items by Rasch Analysis.

(2) Pediatric Balance Scale (PBS)

- Balance control was assessed using the Pediatric Balance Scale (PBS) consisting of 14 items, 0–4 points per item, and a maximum score of 56 points.

(3) Selective Control Assessment of the Lower Extremity (SCALE)

- Selective voluntary motor control capacity was quantified using the Selective Control Assessment of the Lower Extremity (SCALE)
- SCALE score was obtained by summing the 0–2 points assigned to each of the five joints for a maximum of 10 points per limb. For both assessments, higher scores indicated better functioning.

(4) 6 minute walking test and oxygen consumption test

- To assess walking endurance and physical fitness, walking distance (m) and oxygen consumption were measured for 6 minutes using open-circuit spirometry (Kb42, COSMED USA). Subsequently, the oxygen and oxygen costs were calculated.

(5) Bioelectric impedance analysis (BIA)

- BIA was used to estimate muscle mass and body fat percentage.

(6) Pediatric Evaluation of Disability Inventory Computer Adaptive Test (PEDI-CAT)

- measure functional skills in four domains, including daily activities, mobility, social/cognitive, and responsibility
- comprehensive functional assessment to quantify function and measure change after interventions in children with disability
- utilizes a computer adaptive platform with 276 items based on the parent or caregiver report

(7) Computerized gait analysis

- The participants walked barefoot along a 10-m walkway at a self-selected speed. Kinematic data and spatiotemporal gait parameters, including cadence, walking speed (m/s), stride and step length and width, and single and double supports (% of the gait cycle), were measured using a computerized optoelectric motion analysis system (VICON MX-T10 Motion Analysis System, Oxford Metrics Inc., Oxford, UK). The Gait Deviation Index (GDI) was derived from kinematic data.

d. Number of study visits, procedures and duration of each visit: There will be 3 study observation visits at each program for baseline and follow up.

1) pre-intervention
2) Immediately after intervention
3) 4 weeks after end of intervention

baseline (within 72 h before the intervention), at the end of the 6-week intervention (within 1 week after intervention, post-test 1), and after the 4-week follow-up (4±1 week after intervention, post-test 2) to investigate effect maintenance.

e. How long will each participant be involved in the study: Each participant will be involved in the study for at least one week at baseline and one week at follow up. Initial contact with parents/guardians will begin at least one week prior.

f. blinding

To avoid assessment bias, all assessments were completed by physical therapists blinded to the assignment of participants.

7. Costs/Payments:

a. **Costs to participants:** There will be no additional costs to participants.
b. **Type of payment to participants:** Child participants will not be paid.

8. Safety Assessment:

a. **Adverse event (AE) reporting plan including frequency and who will be responsible:**

The Principal Investigator will report any adverse events promptly.

b. Data safety and monitoring plan including data reviewed, frequency, and who will be responsible: N/A

9. Study Monitoring and Quality Assurance:

a. **Protocol adherence monitoring plan including data reviewed, frequency, and who will**

be responsible: The Principal Investigator will closely monitor data after data collection

to ensure the information is collected according to protocol.

10. Privacy and Confidentiality:

1. **Methods used to protect the privacy of participants:** Participants and their parent/guardian will be given the choice to participate in this study. Parents/guardians will provide consent, and children will provide assent. Site director's written consent for interviews, questionnaires, and the observational assessment will be obtained from consent forms distibuted by study staff during program visits.

2. **Methods used to protect the confidentiality of data collected:** Individual data will be seen only by study staff. Summarized results at the level of the program group will be shared with program staff, but individual data will not be shared. No identifying information will be collected. Study staff will maintain a program roster in order to keep track of consent/assent. An arbitrary unique participant ID will be matched to the each data, and analyses will be conducted using this participant ID as the unit of analysis. No names will be used.

Statistical Analysis Plan

Data Management

All data will be stored securely at the centralized web-based system for multicenter trial. Personal identifiers will be kept separately.

Data Analysis Software

SPSS version 28 (IBM Corp., Armonk, NY, USA).

Data Analyses

The Mann–Whitney test, chi-square test, or Fisher's exact test will be us ed to compare the baseline demographic characteristics of both groups ac cording to the normality of the variables. A linear mixed-effects regressio n model will be used to compare the efficacy of RAGT with that of conve ntional PT. The Mann–Whitney U test or independent t-test will be used t o compare the extent of improvement between the baseline and post-inter vention statuses.
