# Supplementary material for: Overground Gait Training With a Wearable Robot in Children With Cerebral Palsy: A Randomized Clinical Trial
Source: JAMA Netw Open. 2024 Jul 22;7(7):e2422625. doi: 10.1001/jamanetworkopen.2024.22625 (PMC11265136; doi:10.1001/jamanetworkopen.2024.22625)
Supplement: Supplement 2. — eFigure 1. The Torque-Assisting Wearable Exoskeletal Robot eFigure 2. Study Flowchart eAppendix. Conventional Physical Therapy (PT) for Control Group [file jamanetwopen-e2422625-s002.pdf]

Supplementary Online Content

Choi JY, Kim SK, Hong J, et al. Overground gait training with a wearable robot in children with cerebral palsy: a randomized clinical trial. *JAMA Netw Open*. 2024;7(7):e2422625. doi:10.1001/jamanetworkopen.2024.22625

eFigure 1. The Torque-Assisting Wearable Exoskeletal Robot

eFigure 2. Study Flowchart

eAppendix. Conventional Physical Therapy (PT) for Control Group

This supplementary material has been provided by the authors to give readers additional information about their work.

© 2024 Choi JY et al. *JAMA Network Open.*

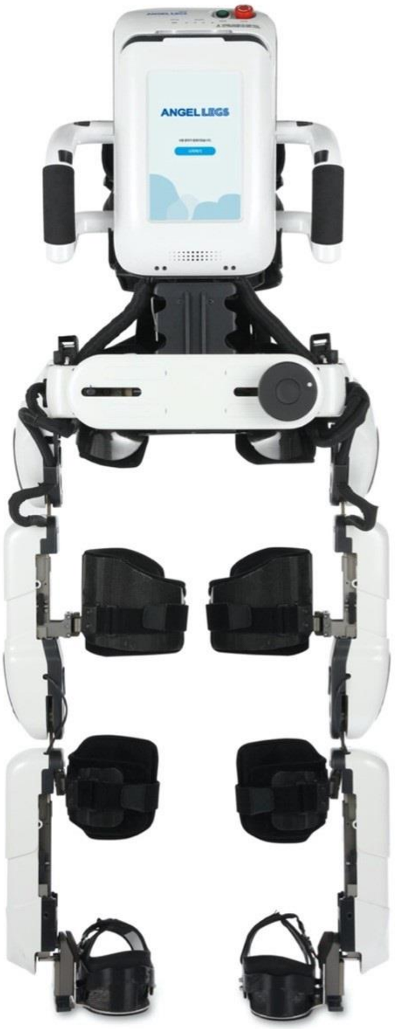

eFigure 1. The torque-assisting wearable exoskeletal robot, Angel Legs M20 (ANGEL Robotics co., Ltd., Seoul, Korea). Child-sized M20 dimensions: 390-510mm [W] x 370-435mm [D] x 1170-1430mm [H]; weight, 14.2kg).

© 2024 Choi JY et al. *JAMA Network Open.*

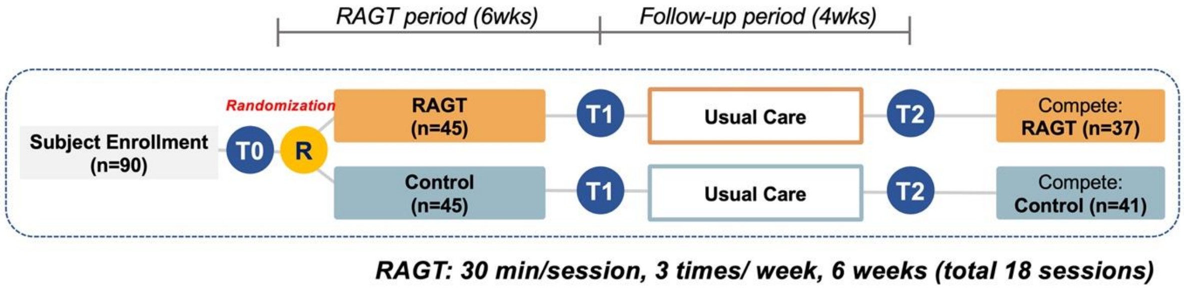

eFigure 2. Study flow chart
RAGT, robot-assisted gait training; T0, baseline assessment; T1, post-intervention; T2, follow-up after 4 weeks assessment.

© 2024 Choi JY et al. *JAMA Network Open.*

eAppendix. Conventional Physical Therapy for Control Group

The control group received conventional physical therapy (PT) focused specifically on gait training. This therapy was administered in a one-to-one setting to ensure personalized attention and tailored intervention for each participant.

Protocol Details:

1. **Frequency and Duration:** Each participant attended PT sessions three times a week, with each session lasting 30 minutes.
2. **Therapy Focus:** The main emphasis was on improving walking abilities through specific exercises that target gait training, strength, and balance.
3. **Therapist Qualifications:** All sessions were conducted by licensed physical therapists specialized in pediatric rehabilitation, particularly in managing cerebral palsy.
4. **Adaptations and Modifications:** Therapy was adapted based on individual progress and specific needs, with modifications made to optimize outcomes.

© 2024 Choi JY et al. *JAMA Network Open.*
