# Supplementary material for: Overground Gait Training With a Wearable Robot in Children With Cerebral Palsy: A Randomized Clinical Trial
Source: JAMA Netw Open. 2024 Jul 22;7(7):e2422625. doi: 10.1001/jamanetworkopen.2024.22625 (PMC11265136; doi:10.1001/jamanetworkopen.2024.22625)
Supplement: Supplement 3. — Data Sharing Statement [file jamanetwopen-e2422625-s003.pdf]

Data Sharing Statement

Choi. Overground Gait Training With a Wearable Robot in Children With Cerebral Palsy. *JAMA Netw Open*. Published July 22, 2024. doi:10.1001/jamanetworkopen.2024.22625

Data
Data available: No

Additional Information
Explanation for why data not available: Anonymized participant data will be shared after approval by the corresponding author, following a reasonable submitted request. MKS and JYC had full access to all the data in the study and takes responsibility for the integrity of the data and the accuracy of the data analysis.
